# Scaffolds and Surfaces with Biomedical Applications

**DOI:** 10.3390/polym15092126

**Published:** 2023-04-29

**Authors:** Andrada Serafim, Stefan Ioan Voicu

**Affiliations:** Advanced Polymers Materials Group, University Politehnica of Bucharest, Gheorghe Polizu Str. 1-7, 011061 Bucharest, Romania; andrada.serafim0810@upb.ro

The engineering of scaffolds and surfaces with enhanced properties for biomedical applications represents an ever-expanding field of research that is continuously gaining momentum. As technology and society evolve, the golden standard of autografts has been contested due to their lack of availability, and tremendous efforts have been dedicated to developing nature-inspired materials that are able to either undertake the functions of damaged tissues or significantly contribute to their repair. To this end, multidisciplinary research that aims to design novel or upgraded materials has been conducted with the purpose of locating suitable candidates that replicate the characteristics of natural tissues with regard to their function, mechanical behavior, microarchitectural features, etc. The present Special Issue entitled ‘Scaffolds and Surfaces with Biomedical Applications’ published 13 papers (10 research and 3 review papers) that describe the synthesis of new materials with biomedical applications and their thorough characterization using conventional and emerging techniques. 

Istratov et al. [1] synthesized new biodegradable implants, starting with the polymerization of L-lactide, catalyzed by tin (II) 2-ethylhexanoate in the presence of 2,2-bis(hydroxymethyl)propionic acid, and an ester of polyethylene glycol monomethyl ester and 2,2-bis(hydroxymethyl)propionic acid; these were accompanied by the introduction of a pool of hydrophilic groups, which minimize the contact angle that leads to the formation of branched pegylated copolylactides with a narrow molecular weight distribution.

Soria et al. evaluated the cytotoxic capacity of a new instillation technology via a biodegradable ureteral stent/scaffold coated with a silk fibroin matrix for application in the controlled release of mitomycin C as an anti-cancer drug [2]. They concluded that the coating of a biodegradable ureteral stent with a silk fibroin matrix impregnated in layers of mitomycin C allows the release of the cytostatic into artificial urine; this finding is crucial to the treatment of patients with a urothelial tumor of the upper urinary tract.

A 3D scaffold structure, comprising thermoplastic polyurethane and maghemite (*ϒ*-Fe_2_O_3_) nanoparticles mixed with ultra-hard and tough bio-resin, was reported by Fallahiarezoudar et al. [3]. Lastly, the presence of ϒ-Fe_2_O_3_ in the structure enhanced the proliferation rate of HSF1148 due to the ability of numerous magnetic nanoparticles to integrate with the cellular matrix.

Filipov et al. [4] presented the development of antimicrobial surfaces that combat implant-associated infections and simultaneously promote the host cell response; this was performed in order to enhance current therapies for orthopedic injuries. The authors reported the modification of 3D-printed polycaprolactone scaffolds with a femtosecond laser and their applicative potential in the production of patterns that resemble microchannels or microprotrusions. The parallel microchannels enabled successful guidance and enhanced the osteogenic potential of MG63 cells. In combination with the improved cytocompatibility, the same microtopography exhibited potent antibacterial effects against *S. aureus*. By developing a biodegradable scaffold that has the potential to simultaneously promote bone tissue regeneration and prevent bacterial biofilm formation, we come a step closer to overcoming the current problems encountered in bone tissue engineering.

Olaret et al. [5] proposed a simple method that can be utilized to obtain nanostructured hydrogels with enhanced mechanical characteristics and relevant antibacterial behavior for application in articular cartilage regeneration and repair; this method was based on low quantities of silver-decorated carbon-nanotubes that were used as reinforcing agents in the semi-interpenetrating polymer network. The main challenge encountered when utilizing hydrogels in applications related to tissue regeneration is represented by their inadequate mechanical properties; although hydrogels are usually soft, most of them are not able to withstand high values of stress. This research aimed to design nanocomposites that exhibit both elasticity and toughness by simultaneously employing two distinct approaches: (1) embedding the linear polyacrylamide in the 3D network of the corresponding monomer and cross-linker with the aim of improving the dispersion of carbon nanotubes in the precursor and the scaffolds’ elasticity, and (2) using low ratios of nanoparticles as fillers, with the aim of providing toughness to the obtained nanostructured system.

Morales-Guadarrama et al. [6] reported the ability of diffusion tensor imaging to evaluate the evolution of spinal cord injuries in nonhuman primates via a fraction of anisotropy analysis and the diffusion tensor tractography calculus. The results revealed that the interrupted nerve fibers can be differentiated from intact regions and that the method can be applied as a qualitative indicator of spinal cord injury in order to represent nerve fibers and to observe the spinal cord evolution after an injury.

Hu et al. [7] evaluated the adult human smooth muscle cell release of angiogenic/growth factor-enriched exosomes in the coronary artery when cultured on *Bombyx mori* 3D silk fibroin nonwoven scaffolds in vitro.

In addition, Neto et al. [8] reported the development of multifunctional biphasic calcium phosphate scaffolds coated with two biopolymers—poly(ε-caprolactone) or poly(ester urea) —loaded with the antibiotic drug Rifampicin, which possesses antibacterial properties, for application in bone regeneration.

Martinez-Garcia et al. [9] presented the synthesis of hydrogels based on the decellularized proteins and polysaccharides of the extracellular matrix that can replicate in vivo functions. Their results revealed that the elasticity of extracellular matrix hydrogels, but also their viscoelastic relaxation and gelling behavior, are organ dependent. Some these physical features are correlated with their biochemical composition and ultrastructure.

Lu et al. [10] presented a facile fabrication method for application in the protection of respiratory masks via electrospinning and the utilization of a nontoxic polyvinyl butyral polymeric matrix with the antibacterial component Thymol, a natural phenol monoterpene. Based on the results of Japanese Industrial Standards and American Association of Textile Chemists and Colorists methods, the maximum antibacterial values of the mask against Gram-positive and Gram-negative bacteria were 5.6 and 6.4, respectively.

This Special Issue also contains three review papers. The first, contributed by Arifin et al. [11], one aims to review the applicative potential of the photo-polymerization 3D printing technique in the fabrication of tissue engineering scaffolds. The review also highlights the comprehensive comparative study of photo-polymerization 3D printing and other scaffold fabrication techniques. Various parameter settings that influence mechanical properties, biocompatibility and porosity behavior are also discussed in detail. The second review paper, contributed by Radu et al. [12], is related to functionalized polysulfone membranes with enhanced hemocompatibility and applicative potential in hemodialysis. The final review paper, reported by Mustafa et al. [13] provides an overview of the utilization of a computational method in designing a unit cell of a bone tissue engineering scaffold.

## Data Availability

Not applicable.
